# Multiparametric magnetic resonance imaging predicts clinical outcomes in patients with chronic liver disease

**DOI:** 10.1016/j.jhep.2015.10.009

**Published:** 2016-02

**Authors:** Michael Pavlides, Rajarshi Banerjee, Joanne Sellwood, Catherine J. Kelly, Matthew D. Robson, Jonathan C. Booth, Jane Collier, Stefan Neubauer, Eleanor Barnes

**Affiliations:** 1 Translational Gastroenterology Unit, University of Oxford, UK; 2 Oxford Centre for Clinical Magnetic Resonance Research, Division of Cardiovascular Medicine, Radcliffe Department of Medicine, University of Oxford, UK; 3 Perspectum Diagnostics, Oxford, UK; 4 Royal Berkshire Hospital, Reading, UK; 5 Peter Medawar Building, University of Oxford, Oxford, UK

**Keywords:** MR, magnetic resonance, LIF, Liver Inflammation and Fibrosis score, NAFLD, non-alcoholic fatty liver disease, NASH, non-alcoholic steatohepatitis, ELF, Extended Liver Fibrosis, MRE, magnetic resonance elastography, ^1^H-MRS, proton magnetic resonance spectroscopy, HCC, hepatocellular carcinoma, cT_1_, iron corrected T_1_, LMS, LiverMultiScan, T_1_ mapping, Iron corrected T_1_, LIF score, LiverMultiScan

## Abstract

**Background & Aims:**

Multiparametric magnetic resonance (MR) imaging has been demonstrated to quantify hepatic fibrosis, iron, and steatosis. The aim of this study was to determine if MR can be used to predict negative clinical outcomes in liver disease patients.

**Methods:**

Patients with chronic liver disease (n = 112) were recruited for MR imaging and data on the development of liver related clinical events were collected by medical records review. The median follow-up was 27 months. MR data were analysed blinded for the Liver Inflammation and Fibrosis score (LIF; <1, 1–1.99, 2–2.99, and ⩾3 representing normal, mild, moderate, and severe liver disease, respectively), T_2_∗ for liver iron content and proportion of liver fat. Baseline liver biopsy was performed in 102 patients.

**Results:**

Liver disease aetiologies included non-alcoholic fatty liver disease (35%) and chronic viral hepatitis (30%). Histologically, fibrosis was mild in 54 (48%), moderate in 17 (15%), and severe in 31 (28%) patients. Overall mortality was 5%. Ten patients (11%) developed at least one liver related clinical event. The negative predictive value of LIF <2 was 100%. Two patients with LIF 2–2.99 and eight with LIF ⩾3 had a clinical event. Patients with LIF ⩾3 had a higher cumulative risk for developing clinical events, compared to those with LIF <1 (*p* = 0.02) and LIF 1–1.99 (*p* = 0.03). Cox regression analysis including all 3 variables (fat, iron, LIF) resulted in an enhanced LIF predictive value.

**Conclusions:**

Non-invasive standardised multiparametric MR technology may be used to predict clinical outcomes in patients with chronic liver disease.

## Graphical abstract

**Figure:**
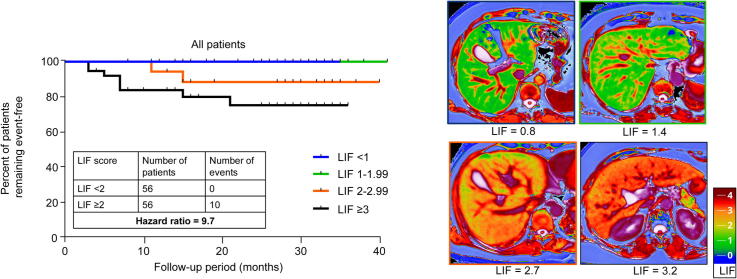

## Introduction

Liver disease in Western populations has reached epidemic proportions, where a third of all adults have some degree of non-alcoholic fatty liver disease (NAFLD) [1]. The estimates for the prevalence of non-alcoholic steatohepatitis (NASH), the more aggressive form of NAFLD, are as high as 12% [2]. Furthermore, viral hepatitis affects over 450 million people worldwide [3], [4], and is associated with cirrhosis in 20% of this population. After developing cirrhosis, patients may remain well (“compensated”) for long periods of time, but approximately 5–7% will develop complications (become “decompensated”) annually. Furthermore, annual mortality rates can be as high as 57% once cirrhosis is established [5]. In the face of this epidemic, there is an urgent clinical need for technologies that can identify patients with chronic liver disease, and risk stratify those who will develop complications or die from liver disease. This will facilitate timely therapeutic interventions, liver transplantation, and stratification within clinical studies.

Traditionally, clinicians have used needle biopsy to assess liver fibrosis. However, as this procedure is painful, requires hospitalisation for several hours or more, and is associated with a risk of complications and death, non-invasive methods for liver fibrosis assessment have been developed in the last decade. Furthermore, liver biopsy is associated with both sampling and observer dependent variability [6], [7]; therefore the use of this as a gold standard comparator for the development of non-invasive technologies is sub-optimal [8]. A more robust and clinically relevant approach would involve the assessment of whether non-invasive technologies can be used to predict clinically meaningful endpoints.

Broadly, non-invasive techniques can be divided into those based on direct and indirect serum markers of fibrosis and those based on imaging and or elastography. Serum biomarkers are attractive as they are easy to measure and can be repeated over time. However, they lack specificity as they may be affected by extrahepatic fibrosis. For example, the Extended Liver Fibrosis (ELF) panel reported a sensitivity of 90%, but had a specificity of only 41%, with an area under the receiver operating characteristic curve of 0.80 for the detection of severe fibrosis [9]. A subsequent 7 year follow-up study suggested that ELF score could predict clinical outcomes [10]. However, patients with any disorder associated with extrahepatic fibrosis were excluded from these studies making it difficult to assess how this test could be applied to a general, unselected population.

Liver stiffness measurement using magnetic resonance elastography (MRE), ultrasound-based transient elastography (Fibroscan™), or acoustic radiation force impulse have also been used to assess fibrosis and for predicting clinical outcomes [11], [12], [13], [14], [15]. However, ultrasonic elastography cannot be used if there is significant fat or fluid between the chest wall and the liver; failed readings or unreliable results are observed in nearly 20% of patients, particularly those with obesity and the metabolic syndrome [16]. Furthermore, elastography measures have been shown to carry considerable variance [17]. MRE is more accurate than transient elastography [18], but needs additional hardware and is compromised in patients with haemosiderosis.

Overall, despite these advances in non-invasive liver assessment, the drawbacks of the currently available techniques mean that they are not widely available and have not been validated for use as surrogate endpoints in clinical trials. Because of this, several professional and regulatory bodies recognise the need for better stratification tools [19], [20], [21].

Magnetic resonance (MR) techniques offer an attractive non-invasive option for liver assessment. These are well established in assessing anatomical morphology, are organ specific and have the capacity to evaluate the whole organ thereby eliminating all the concerns around sampling error. Furthermore, they can be standardised across scanner vendors and magnet strengths so that inter-operator variability is negligible.

T_1_ mapping is a MR technique that allows *in vivo* tissue characterisation. At our centre, a multiparametric MR technique has been established, that includes T_1_ mapping for fibrosis/inflammation imaging, T_2_∗ mapping for liver iron quantification and proton magnetic resonance spectroscopy (^1^H-MRS) for liver fat quantification. The T_1_ measurements in our method are adjusted for the iron level, as high iron levels in the presence of fibrosis can lead to “pseudo-normal” T_1_ values. This is a quick and truly non-invasive test that does not require injection of any intravenous contrast agent. In a recent study, it has shown good correlation with histological parameters in a cohort of patients with mixed liver disease aetiologies undergoing clinically indicated liver biopsy [22].

The aim of the present study was to assess whether data obtained from this multiparametric MR protocol could be used to predict all-cause mortality and liver related clinical events, irrespective of stage of fibrosis or disease aetiology.

## Patients and methods

### Study design and patient population

**Fig. 1:**
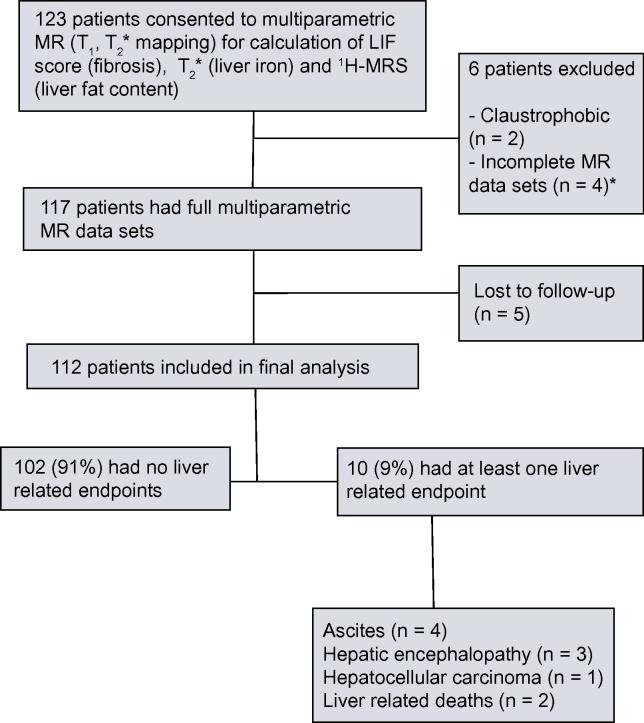
**Study flow chart.** The Liver Inflammation and Fibrosis (LIF) score is a standardised continuous score (0–4) derived from liver T_1_ and T_2_∗ values. T_1_ primarily reflects the amount of extracellular fluid and can change with inflammation and fibrosis and T_2_∗ primarily reflects the amount of iron deposition. Liver iron has a confounding effect on T_1_, and this is accounted for in the LIF score calculation. ^∗^Liver iron concentration from T_2_∗ maps and hence LIF calculation was not possible in 4 cases. MR, magnetic resonance; ^1^H-MRS: proton (^1^H) magnetic resonance spectroscopy; LIF, Liver Inflammation and Fibrosis score.

The population under study were those scheduled to have a clinically indicated liver biopsy (n = 116) and adult patients who had cirrhosis diagnosed on biopsy within 5 years of their MRI scan (n = 7). Patients were included irrespective of underlying liver disease aetiology or disease stage. The only exclusion criterion was the presence of MRI contraindications. Patients were recruited from two UK centres (Oxford and Reading) between April 2011 and August 2013. All patients were followed for the development of clinical outcomes except those who were lost to follow-up or had incomplete MR data (n = 11; Fig. 1). Baseline data were collected at the time of recruitment. Outcome data were collected through review of the individual electronic and paper patient records.

The study was approved by the UK National Research Ethics Service and the institutional review board and was performed in accordance with the ethical guidelines of the 1975 Declaration of Helsinki. All patients gave written informed consent.

### All-cause mortality and liver related clinical events

Both all-cause mortality and liver related clinical events were evaluated. Liver related clinical events were defined as liver related death, the development of hepatocellular carcinoma (HCC) and any new episode of hepatic decompensation (clinically evident ascites, variceal bleeding, and hepatic encephalopathy). Although more than one event per patient was possible and occurred, patients were only counted once in the analysis at the time of the first liver related clinical event. Patients who had evidence of liver related complications at or before enrolment were only counted again if they developed a complication that was distinct from that observed before enrolment, or died. Patients were followed up until their last clinical review or until they died, but the index liver related event as defined above was used in the analysis.

### Multiparametric MRI

The MRI technique used in this study has been previously described [22]. Briefly, MR scans were performed in Oxford using a 3-Tesla scanner (Tim Trio, Siemens Healthcare, Germany). Transverse abdominal T_1_ and T_2_∗ MR maps were acquired for the estimation of extracellular fluid and liver iron respectively. Proton magnetic resonance spectroscopy (^1^H-MRS) was also used to measure liver fat content. Patients attended for their MRI scans after fasting for at least 4 h.

### Iron corrected T_1_ and the Liver Inflammation and Fibrosis score

T_1_ relaxation time increases with increases in extracellular fluid such as in fibrosis and inflammation. However, the presence of iron, which can be accurately measured from T_2_∗ maps, has an opposing effect on the T_1_. An algorithm has been created that allows for the bias introduced by elevated iron to be removed from the T_1_ measurements, yielding the iron corrected T_1_ (cT_1_). This has been described in detail previously [22], [23]. This correction was necessary in 54% of patients in this study that were found to have a liver T_2_∗ <19 ms, corresponding to a dry weight liver iron concentration of >1.3 mg/g, indicating some degree of iron overload, which affects T_1_ measurements.

Optimal cT_1_ cut-off points for the differentiation of: no (Ishak fibrosis stage F0), mild (Ishak F1–2), moderate (Ishak F3–4), and severe (Ishak F5–6) fibrosis have been derived from the association of cT_1_ with histological fibrosis in our previous study [22]. These cut-offs were used to develop the Liver Inflammation and Fibrosis (LIF) score, a standardised continuous score (0–4) which can be derived from many MR systems. For this study, patients were categorised according to LIF score into having: no (LIF <1), mild (LIF 1–1.99), moderate (LIF 2–2.99), or severe (LIF 3–4) liver disease.

**Fig. 2:**
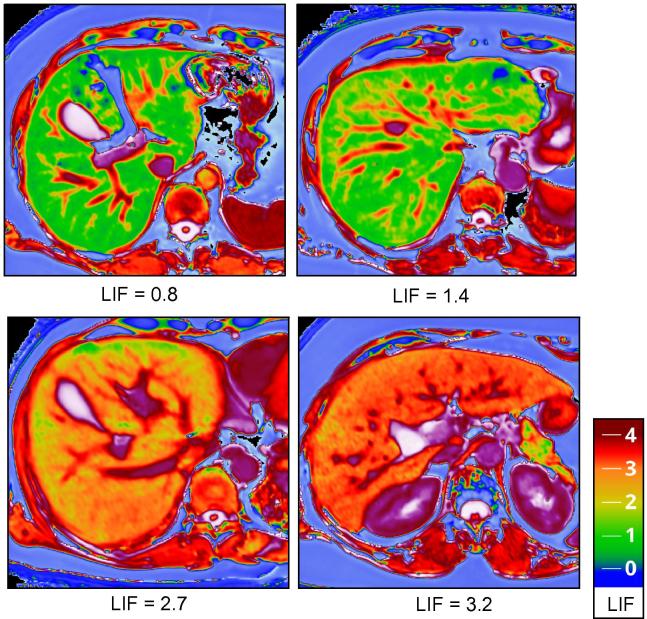
**Examples of LiverMultiScan MR data.** Representative images from patients in each Liver Inflammation and Fibrosis (LIF) severity category, produced by analysis of the raw data using LiverMultiScan. LIF was measured in operator chosen regions of interest in the right liver lobe, in the liver parenchyma, away from vascular and biliary structures. The LIF scores measured for each image in this figure are indicated under each image. The predefined colour scale used to generate these maps is also included. LIF, Liver Inflammation and Fibrosis score. (This figure appears in colour on the web.)

LiverMultiScan™ (LMS, Perspectum Diagnostics, Oxford, UK), is a software product, developed specifically to measure cT_1_ and LIF scores from T_1_ and T_2_∗ maps. For this study, LMS was used to analyse anonymised images off-site, by investigators blinded to the clinical and histological data. LIF scores were measured in a single, operator-defined, region of interest in the right liver lobe, away from vascular and biliary structures. Representative LMS images are shown in Fig. 2.

### Statistical analysis

The primary variable of interest was the LIF score measured using LMS. Patients were stratified according to LIF severity as described above. Secondary variables were liver iron measured by T_2_∗ and liver fat measured by ^1^H-MRS. Cut-off values derived from our previous study [22] were used to define two severity categories for iron (high liver iron: T_2_∗ ⩽12.5 ms, low liver iron: T_2_∗ >12.5 ms) and 3 categories for fat fraction (0: fat <1.5%, 1: fat 1.5– <7.5%, 2: fat ⩾7.5%). The cut-off for defining the iron severity categories represents the best threshold for identifying those with any grade of histological iron deposition and corresponds to a dry weight iron 1.7 mg/g. The cut-offs for defining the liver fat categories represent the best thresholds for identifying those with no steatosis (fat fraction <1.5%) and those with steatosis in more than 66% of hepatocytes (steatosis grade 3; fat fraction ⩾7.5%). The primary outcome was the index liver related event as described above (liver related death, HCC, clinically evident ascites, encephalopathy, variceal bleeding). Subgroup analysis including only patients with compensated disease at baseline was also carried out.

Descriptive statistics were used to summarise the subjects’ baseline characteristics for different LIF categories. The Shapiro–Wilks test was used to test for normality of the data across the whole cohort. Analysis of variance was used to test for differences between multiple groups. Kaplan–Meier analysis was used to establish the proportions of patients in each LIF, fat and iron severity category that remained free of liver related events in the follow-up period. Differences between the curves were compared using the log-rank test. Cox regression analysis was used to examine the additive effects of LIF, liver fat and liver iron (measured by T_2_∗) on predicting liver related events. The level of statistical significance was set at *p* <0.05. The analysis was carried out using SPSS v22 (Armonk, NY; IBM Corp) software. The GraphPad Prism software (v6.04) was used to generate the Kaplan–Meier curves for the illustration in the figures.

## Results

### Baseline characteristics

During the study period 123 patients consented to take part. Six were excluded due to claustrophobia (n = 2) or incomplete MR data (n = 4). Follow-up data were available for 112 of the 117 patients with complete MR data sets (Fig. 1). Patients were lost to follow-up either because they moved out of the area, or they did not return for any further clinical follow-up after their MR scan. The median (IQR) follow-up period was 27 (15–31) months. The majority of patients were male (n = 71, 79%) and the mean (±SD) age was 51 (±13.8) years. Eight (7%) patients had evidence of decompensation at or prior to enrolment, and 16 (14%) patients received treatment for their underlying disease in the follow-up period (6 patients with chronic hepatitis C virus infection achieved sustained virological response, 7 patients with chronic hepatitis B achieved viral suppression on treatment and three patients with obesity had weight reduction surgery). Baseline clinical, histological, and biochemical characteristics for the whole cohort and for the four LIF severity categories are summarised in Table 1.

Liver biopsy was performed in 102 patients. All patients had evidence of chronic liver disease. Chronic viral hepatitis and NAFLD were the two most common histological diagnoses affecting 34 (30%) and 39 (35%) patients, respectively. Histologically, liver fibrosis was assessed using the Ishak score [24], and staged as mild (F0–2) in 54 (48%), moderate (F3–4) in 17 (15%), and severe (F5–6) in 31 (28%) patients.

When patients were stratified using the LIF score; 38 (34%) had severe disease, 18 (16%) had moderate disease, 34 (30%) had mild disease, and 22 (20%) had no liver disease. More patients with high LIF scores had severe histological fibrosis (LIF 3–4: 50%, LIF 2–2.99: 44%, LIF 1–1.99: 12%, LIF <1: 0%, *p* <0.001). Furthermore, patients with high LIF scores had higher BMI (LIF 3–4: 29.2 kg/m^2^, LIF 2–2.99: 27.1 kg/m^2^, LIF 1–1.99: 26 kg/m^2^, LIF <1: 27.1 kg/m^2^, *p* = 0.02), higher liver fat content (LIF 3–4: 17.6%, LIF 2–2.99: 7.1%, LIF 1–1.99: 8.2%, LIF <1: 3.5%, *p* = 0.01) and were more likely to have type 2 diabetes (LIF 3–4: 24%, LIF 2–2.99: 28%, LIF 1–1.99: 6%, LIF <1: 0%, *p* = 0.04).

Seven out of the eight patients who had baseline decompensation had severe disease by LIF criteria (LIF ⩾3) and four of these went on to develop further liver related clinical events. One patient with baseline decompensation (ascites) had mild disease by LIF criteria (LIF 1–1.99) and remained event free during follow-up.

### Follow-up data

Ten (9%) patients developed an index liver related event (4 ascites, 3 encephalopathy, 1 HCC, 2 liver related deaths), and six patients died during the follow-up period. There were no deaths relating to causes other than liver disease. The clinical features of the ten patients who had at least one liver related clinical event are detailed in Table 2.

**Fig. 3:**
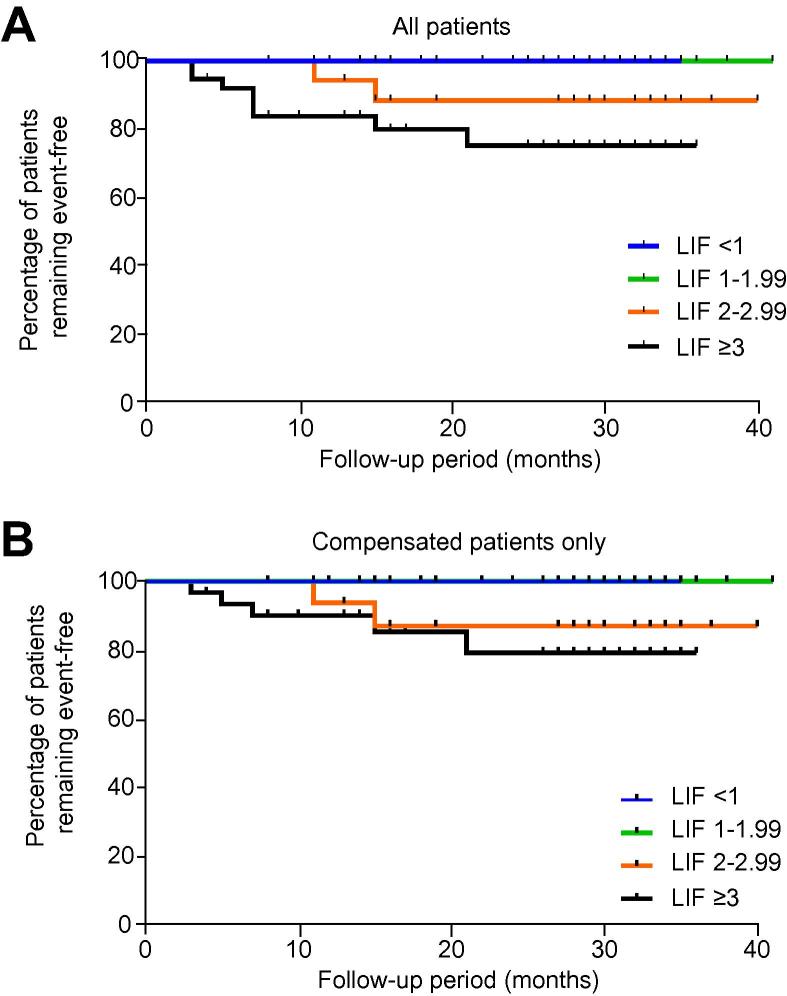
**Kaplan–Meier curves for liver related event free survival with patients stratified according to Liver Inflammation and Fibrosis scores.** In the entire cohort, (A) there were significant differences between those with Liver Inflammation and Fibrosis (LIF) ⩾3 *vs.* LIF <1 (*p* = 0.02) and *vs.* LIF 1–1.99 (*p* = 0.003). There was a strong trend towards significance between LIF 2–2.99 *vs.* LIF 1–1.99 (*p* = 0.054). Including only compensated patients at baseline, (B) there was a significant difference between LIF ⩾ 3 *vs.* LIF 1–1.99 (*p* = 0.023) and a strong trend towards significance between LIF 2–2.99 *vs.* LIF 1–1.99 (*p* = 0.058). (This figure appears in colour on the web.)

There were no liver related events in patients with a LIF score of less than 2 (negative predictive value 100%). Ten out of 56 (18%) patients with a LIF ⩾2 experienced at least one liver related event. Kaplan–Meier analysis showed that patients with a LIF ⩾3 had a significantly higher cumulative risk of developing liver associated clinical complications and death over time, compared to those with LIF <1 (*p* = 0.02) and LIF 1–1.99 (*p* = 0.03). Furthermore, there was a strong trend towards a higher risk of complications in those with LIF 2–2.99 compared to LIF 1–1.99 (*p* = 0.054, Fig. 3A).

When patients with decompensation at or prior to baseline were excluded from the analysis, those with severe disease (LIF 3–4) were still at an increased cumulative risk of complications compared to those with mild disease (LIF 1–1.99; *p* = 0.023), and there was a strong trend for a difference between those with moderate disease (LIF 2–2.99) and those with mild disease (LIF 1–1.99; *p* = 0.058, Fig. 3B).

**Fig. 4:**
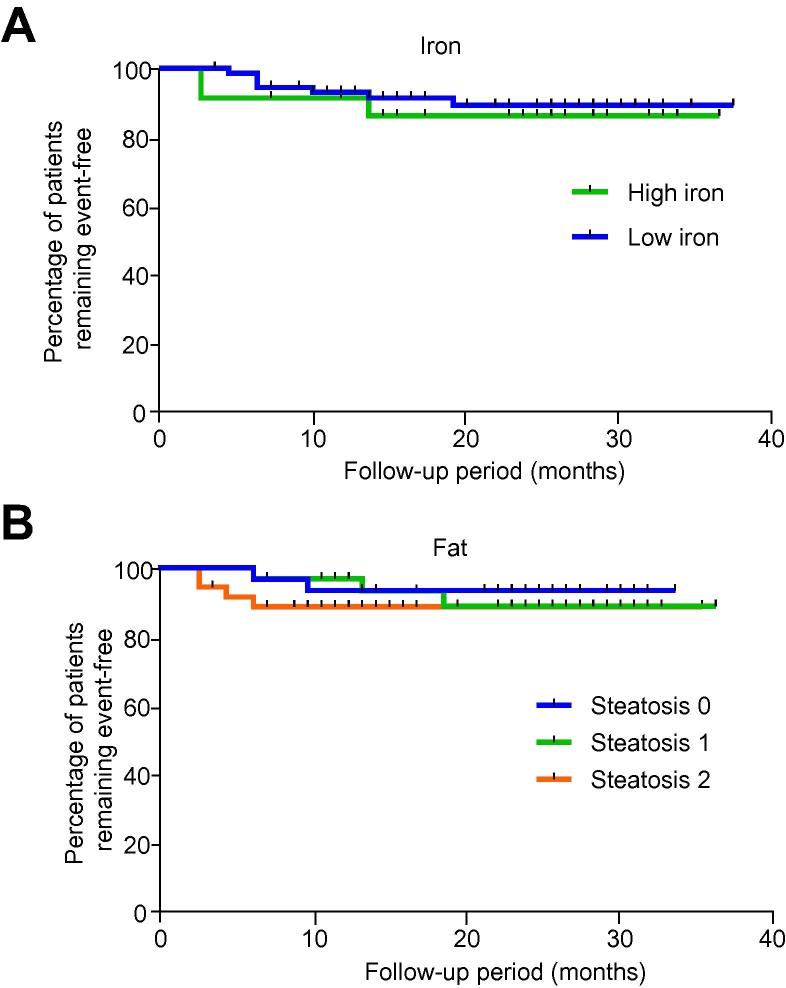
**Kaplan–Meier curves for liver related event free survival with patients stratified according to severity of liver iron and fat.** There were no significant differences between the curves for (A) iron or (B) fat. Liver fat was categorised according to the liver fat content measured by proton magnetic resonance spectroscopy as 0: <1.5%, 1: 1.5% <fat <7.5% and 2: ⩾7.5%. Liver iron was categorised according to T_2_∗ as low iron: T_2_∗ >12.5 ms or high iron: T_2_∗ ⩽12.5 ms. (This figure appears in colour on the web.)

There were no differences in the risk of developing liver related clinical events when patients were stratified according to MR determined severity categories for liver fat and liver iron deposition (Fig. 4).

### Cox regression analysis

LIF had a hazard ratio (HR) of 9.7 (95% CI: 2.2–43.2; *p* = 0.003) when it was entered as the sole variable in the model. The HR increased to 75.7 (95% CI: 7.6–752; *p* <0.001) when all 3 variables were entered into the model. Furthermore, in the model containing all 3 variables liver fat (HR 0.80; 95% CI: 0.70–0.91; *p* <0.001) and T_2_∗ which is inversely related to iron load (HR 0.87; 95% CI: 0.80–0.95; *p* = 0.001), had a protective effect. The results of the Cox regression analysis with all 4 possible covariate combinations including LIF are shown in Table 3.

## Discussion

This study shows that the LIF score, a newly established MR score for the assessment of liver fibrosis and inflammation, strongly predicts clinical outcomes in patients with chronic liver disease of mixed aetiologies. No patients with LIF <2 developed liver related clinical events, in contrast to almost 1 in 5 (18%) of those with LIF ⩾2. Quantification of liver fat and liver iron using a multiparametric MRI technique added to the predictive value of LIF.

This is the first study of liver T_1_ mapping to demonstrate the prognostic value of this technique in a patient population. It follows from our previous work showing that multiparametric MR had good diagnostic accuracy compared to histology, particularly for the diagnosis of early disease, with an area under the receiver operating characteristic curve of 0.94 for the identification of those with any degree of fibrosis [22]. Other studies of similar T_1_ mapping techniques have shown promise in differentiating patients with cirrhosis from normal controls [25], [26], [27].

The enhanced ability of our technique to differentiate between histological stages may be due to the particular quantitative T_1_ mapping technique we apply [28], and the application of a correction algorithm for the confounding effect of liver iron on T_1_ values. The additional finding, that this T_1_ mapping technique may be used to predict negative clinical outcomes, is entirely novel. Our data included patients at all stages of fibrosis and those with known cirrhosis. We specifically included patients with cirrhosis since the presence of cirrhosis on a liver biopsy is simply quantified categorically into present/absent. The advantage of multiparametric MR is that this is able to quantify fibrosis along a numeric continuum, and so stratify patients within the category of cirrhosis into those at high risk of decompensation.

MR-quantified liver fat and liver iron did not provide any useful prognostic information when these parameters were considered in isolation. However, in the multivariate analysis using the Cox regression model, inclusion of fat and iron, added to the predictive value of LIF increasing the HR from 9.7 to 75.7 (Table 3). Furthermore, in the model including all 3 covariates, liver fat and T_2_∗ had a protective effect.

T_2_∗ in inversely related to iron load, therefore the protective effect seen with decreasing liver iron (increasing T_2_∗) would be in keeping with previous observations that increased iron is associated with worse prognosis [29] and more aggressive NAFLD phenotypes [30].

Previous studies have demonstrated that liver fat decreases as fibrosis progresses [31], and also that liver fat is a risk factor for fibrosis progression [32], [33]. The observation here that liver fat is protective, may seem at odds with the published literature. However, the most likely explanation for this finding is that patients with high liver fat would be more likely to have early stages of fibrosis and were therefore at lower risk of disease progression to clinical outcome within the timeframe of this study.

One other important observation from our study was that patients with higher LIF scores had higher BMI, were more likely to have type 2 diabetes and had higher liver fat content. Diabetes, obesity and liver steatosis have all been associated with more severe liver fibrosis [34], [35], [36]. In contrast to what might be expected, in this study 26% of patients with severe disease assessed by MR (LIF ⩾3) and 50% of patients with moderate disease (LIF 2–2.99) had only mild fibrosis using histological staging (Ishak 0–2). The reason for the lack of concordance, between histology and MR parameters in these patients, is not clear. However, whilst this may reflect MR limitations it is equally plausible that histology under-staged the fibrosis severity or that these patients had an inflammatory burden that is not adequately assessed by histology. Further work and longer follow-up is needed to establish whether patients with mild histological fibrosis but high LIF (⩾2) scores are at increased risk progression and decompensation compared to patients with mild histological fibrosis and low LIF (<2) scores.

It is well documented that the lack of reliable surrogate endpoints is impacting on drug development for liver disease. Currently, clinical trials generally rely on histological assessment of fibrosis requiring multiple biopsies over short time frames. This is frequently unacceptable to patients so limiting recruitment, whilst concurrently the sampling and observer dependent variability of liver biopsy will increase the number of patients required in a study to achieve adequate statistical power to meet the study endpoints. Furthermore in clinical practice, liver biopsies are usually repeated every 3–5 years to assess disease progression in patients with known chronic liver disease in order to risk stratify patients and prioritise treatments. This has cost implications both for health service providers and patients.

In contrast to liver biopsy, we have previously shown that measurement of MR parameters is highly reproducible with a coefficient of variance of 1.8% for cT_1_, 8.4% for T_2_∗ and 4.8% for hepatic lipid content measured by ^1^H-MRS, when subjects were scanned twice on the same day [22]. In comparison, a study of MRE reproducibility has shown that 8.2% of the variability in the measured liver stiffness was accounted for by the difference between examinations on the same day [37]. The results however, are not directly comparable as different methods for the assessment of reproducibility were used in the two studies.

In addition, iron corrected T_1_, T_2_∗ and liver fat content measured by ^1^H-MRS, are associated with their respective histological scores [22]. Furthermore, sampling variability is minimised through sampling of large regions of interest, and imaging the whole liver is possible. Data from the whole organ could be captured and integrated if necessary. Lastly, this is a truly non-invasive and quick test that could allow repeated measurements over time and can be standardised across scanner types and magnet strengths. Therefore, similar to other MR biomarkers (MRE, MRI-proton density fat fraction) [38], non-invasive, standardised multiparametric MR could be used as a surrogate endpoint in clinical trials, and as a clinical tool. To this effect we have demonstrated the ability of the LIF score to predict hard clinical outcomes.

This was a small proof of principle study that included patients across a spectrum of liver disease severity and aetiologies. One limitation was the relatively short follow-up duration. However, in spite of this, we were able to demonstrate a utility for multiparametric MR in defining prognosis and the development of clinical endpoints. These results therefore support the further evaluation of multiparametric MR in specific patient populations over longer follow-up periods. Since the majority of patients included in this study were ones that had been referred for liver biopsy by their clinical team, those with clinically evident cirrhosis, and those with no clinical suspicion for significant liver disease may be under-represented.

Due to the relatively short follow-up, only patients with liver cirrhosis developed complications. The inclusion of all patients in the main analysis irrespective of the presence of decompensation at baseline may have biased the results in the overall cohort. However, when patients with clinically evident liver decompensation were excluded from the analysis, statistically significant differences still remained between the LIF severity groups. Furthermore, seven out of the ten patients that developed complications were classified as Child-Pugh class A cirrhosis at baseline, suggesting that the LIF score may provide valuable additional independent prognostic data in patients with compensated liver disease.

Therefore, of particular interest for future longitudinal studies will be the evaluation of multiparametric MR in primary care settings, in population screening studies of patients at risk of metabolic syndrome and NASH (those overweight and/or with diabetes), as is currently under investigation in the UK Biobank population cohort, and in a larger cohort of patients with liver cirrhosis where non-invasive prognostic tests may me be useful in defining appropriate timelines for liver transplantation and other therapeutic interventions.

In conclusion, we provide the first evidence that rapid, non-invasive multiparametric MRI technology that quantifies LIF score in a standardised test, may provide valuable prognostic information in patients with chronic liver disease. This finding extends the results of our previous study demonstrating that multiparametric MRI can be used to quantify liver fibrosis, iron and steatosis, each known to be a critical component in driving liver pathology. Overall, these data support the further evaluation and development of multiparametric MR as a technology that can be used to stage liver disease, identify patients who will progress to cirrhosis and develop complications of liver disease. This would enable timely clinical interventions like transplantation and drug therapy, and facilitate clinical trials as a robust surrogate endpoint.

## Financial support

The study was supported by grants from the Oxford NIHR Biomedical Research Centre (MP and JS).

## Conflict of interest

MP, RB, CJK, MDR, SN and EB are shareholders in Perspectum Diagnostics, A University of Oxford spin out company. RB, MDR and SN are on the board of directors of Perspectum Diagnostics. RB and CJK are employees of Perspectum Diagnostics. MP, RB, MDR, SN and EB have filed patent applications related to the use of MRI for the assessment of liver disease. JS, JC and JCB have no conflict of interest to declare.

## Authors’ contributions

MP, RB, SN and EB designed the study. MP and RB collected MR data. MP and JS collected outcome data. MP, RB and CJK performed MR data analysis. RB, CJK, MDR and SN designed the LIF score. MP, RB, JC, JCB and EB recruited patients to the study. MP, RB, SN and EB drafted the manuscript. All authors revised the manuscript critically for intellectual content, and have approved the final version.

## Figures and Tables

**Table 1:** **Baseline patient characteristics.**

*p* values quoted for the differences between the 4 LIF severity groups.

^*^One patient in this subgroup had alcoholic hepatitis and cirrhosis on biopsy.

^+^Other included: non-specific cholestasis (n = 4), no specific diagnostic features (n = 2), sarcoid (n = 1), sinusoidal dilatation (n = 1), cryptogenic cirrhosis (n = 1), haemochromatosis (n = 1).

LIF, Liver Inflammation and Fibrosis score; SD, standard deviation; IQR, inter-quartile range; BMI, body mass index; T2DM, type 2 diabetes mellitus; ^1^H-MRS, proton magnetic resonance spectroscopy; NAFLD, non-alcoholic fatty liver disease; ASH, alcoholic steatohepatitis; PSC, primary sclerosing cholangitis; PBC, primary biliary cirrhosis; AIH, autoimmune hepatitis; ALT, alanine aminotransferase; ALP, alkaline phosphatase; GGT, gamma glutamyl transferase; AST, aspartate aminotransferase.

**Table 2:** **Clinical features of patients who had liver related events in the follow up period.**

^*^The patient took part in the imaging study but did not attend his liver biopsy. He had a history of excessive alcohol use suggestive of a diagnosis of alcoholic steatohepatitis.

LIF, Liver Inflammation and Fibrosis score; CP, Child-Pugh class; NAFLD, non-alcoholic fatty liver disease; HCV, hepatitis C; ASH, alcoholic steatohepatitis; HCC, hepatocellular carcinoma.

**Table 3:** **Cox regression analysis for the prediction of liver related events for LIF, fat and T_2_∗.**

LIF, Liver Inflammation and Fibrosis score; HR, hazard ratio, 95% CI: 95% confidence interval.
